# From Infection Control to Tissue Regeneration: Mechanisms, Design Strategies, and Smart Advances in Antibacterial Hydrogels

**DOI:** 10.3390/gels12090812

**Published:** 2026-09-04

**Authors:** Peng Liu, Lin Chen, Jinju Tian, Dan Wang, Yiping Deng, Xiangdi Jia, Zanxia Cao, Mingqiong Tong

**Affiliations:** 1 Experiment Management Centre, Dezhou University, Decheng District, Dezhou 253023, China; 2 Shandong Engineering Research Center of Novel Pharmaceutical Excipients, Sustained and Controlled Release Preparations, College of Health and Medicine, Dezhou University, Decheng District, Dezhou 253023, China; 3 Shandong Engineering Research Center of Novel Pharmaceutical Excipients, Sustained and Controlled Release Preparations, College of Pharmacy, Dezhou University, Decheng District, Dezhou 253023, China; 4 Institute of Biophysics, Dezhou University, Decheng District, Dezhou 253023, China

**Keywords:** antibacterial hydrogels, antibacterial mechanisms, dynamic crosslinking, stimuli responsiveness, tissue repair, integrated diagnosis and therapy

## Abstract

Bacterial infection, biofilm formation, and the associated oxidative stress and persistent inflammation represent major obstacles to wound healing, tissue engineering, and implantable medical devices. Owing to their highly hydrated three-dimensional networks, favorable tissue compatibility, and versatile capacity for functional loading, hydrogels have been widely investigated for the treatment of infected wounds. This review systematically summarizes the major antibacterial mechanisms of hydrogels, including cationic contact-killing, chemical antibacterial activity mediated by metal ions and reactive halogen species, nanozyme-catalyzed reactions and bidirectional regulation of reactive oxygen species, as well as photothermal synergistic antibacterial therapy. Key design strategies are also discussed, including natural polymer-based matrices, multiple dynamic crosslinking, stimuli-responsive controlled release, three-dimensional printing, and spatial compartmentalization. In addition, recent advances in infection-microenvironment regulation, wet-interface adaptation, temporally coordinated tissue repair, and integrated diagnosis and therapy are highlighted. The field is currently shifting from single-mode bacterial eradication toward multistage tissue repair and intelligent theranostics. However, major challenges remain, including balancing antibacterial efficacy with biosafety, achieving reproducible manufacturing and sterilization-compatible formulations, maintaining functional stability during storage, and improving the clinical relevance and standardization of preclinical evaluation. In addition, most smart systems still lack quantitative coupling among pathological signals, therapeutic dosage, and treatment outcomes. Future studies should therefore integrate mechanistic design with manufacturing reproducibility, clinically relevant validation, and quantitative feedback regulation, thereby advancing antibacterial hydrogels from multifunctional proof-of-concept systems toward precise, controllable, and clinically translatable therapeutic platforms.

## 1. Introduction

Bacterial infections and biofilm formation are prevalent in biomedical settings, including wounds, implantable devices, and tissue-engineered materials, where they can cause local tissue damage, persistent inflammation, and treatment failure, thereby substantially complicating infection control [1,2]. Particularly in complex pathological conditions such as diabetic wounds, the interplay among hyperglycemia, hypoxia, oxidative stress, impaired microcirculation, and increased susceptibility to infection sustains an aberrant inflammatory state, disrupts normal tissue repair, and ultimately leads to chronic non-healing wounds [3,4,5,6,7]. Therefore, developing novel therapeutic materials that can coordinate pathogen elimination, infection-microenvironment regulation, and subsequent tissue repair, while maintaining tissue-interface adaptability and biocompatibility, has become an important research focus in infected wound treatment and biomedical materials [8,9,10].

Hydrogels have been widely applied in infected wound healing because of their favorable moisture-retention capacity, biocompatibility, and extracellular matrix-like three-dimensional network structure [11,12,13,14]. Their networks not only enable the loading and sustained release of antibacterial drugs, metal ions, nanomaterials, and bioactive molecules, but can also be engineered through the regulation of polymer composition, crosslinking modes, network architecture, and interfacial chemistry to provide wet adhesion, self-healing, antioxidant, and stimuli-responsive properties. These features facilitate wound coverage and protection, exudate management, and local microenvironment regulation [15,16,17,18,19,20]. However, conventional single-network hydrogels often suffer from insufficient mechanical strength, limited stability under wet conditions, and inadequate control over the release of functional components, making it difficult to meet the combined requirements of effective infection control, microenvironment regulation, and subsequent tissue regeneration in complex infected wounds [21].

Unlike previous reviews that mainly focus on antibacterial agents, hydrogel formulations, or multifunctional integration separately, this review aims to establish an evolutionary framework describing how antibacterial hydrogels transition from pathogen elimination toward coordinated infection microenvironment regulation and tissue regeneration. Specifically, we organize recent advances according to three progressive stages: (1) antibacterial eradication through direct bacterial killing and biofilm disruption; (2) microenvironment remodeling through ROS regulation, immune modulation, and responsive therapeutic release; and (3) intelligent tissue repair through adaptive interfaces, spatially programmed structures, and theranostic feedback systems. Within this framework, antibacterial mechanisms, material/network engineering, and intelligent theranostic functions are regarded not as independent topics, but as interconnected design levels that determine how antibacterial activity is generated, regulated, and ultimately coordinated with tissue repair.

At the first level of this evolutionary framework, effective pathogen elimination remains the prerequisite for subsequent microenvironment recovery and tissue repair. Antibacterial hydrogels have evolved from simple antibiotic-loaded systems into multimodal synergistic platforms. By integrating drug release, nanomaterial-mediated oxidative damage, membrane disruption, and stimuli-responsive regulation, these systems can achieve efficient and sustained antibacterial activity [22]. Specifically, cationic polymers primarily disrupt bacterial membranes through electrostatic interactions; metal ions such as Ag^+^, Cu^2+^, and Zn^2+^ interfere with membrane integrity, protein function, and energy metabolism; N-halamines and nanozymes exert bactericidal effects through reactive halogen-mediated oxidation and catalytic generation of reactive oxygen species (ROS), respectively; and photothermal materials induce bacterial membrane damage and protein denaturation through localized heating [23,24,25,26,27,28]. In addition, hydrogel systems can enhance the accumulation and synergistic action of antibacterial agents at infected sites by incorporating functional components such as natural polyphenols, antibiotics, and photodynamic or photothermal therapeutic agents, thereby improving their inhibitory effects against Gram-positive bacteria, Gram-negative bacteria, and drug-resistant strains [29,30]. However, enhanced antibacterial efficacy is often accompanied by potential safety risks, including cytotoxicity caused by excessive metal-ion release, oxidative damage induced by reactive oxygen species accumulation, and tissue injury resulting from excessive local heating during photothermal therapy [16]. Therefore, the key challenge is not simply to maximize antibacterial efficacy, but to achieve effective pathogen eradication without compromising the subsequent regulation of the wound microenvironment and tissue repair [8].

To translate these antibacterial mechanisms into safe, sustained, and controllable therapeutic effects, the design of antibacterial hydrogels has gradually shifted from simple component integration toward the coordinated engineering of network structures and functional processes. The incorporation of natural polymer frameworks, nanoreinforced architectures, rigid-flexible composite networks, and dynamic reversible crosslinking can markedly improve the mechanical stability, energy-dissipation capacity, self-healing ability, and tissue-interface adaptability of hydrogels [31,32,33,34]. By exploiting endogenous and exogenous stimuli, including pH, reactive oxygen species, enzymes, temperature, and near-infrared light, these systems can sense the infection microenvironment and achieve spatiotemporally controlled release of antibacterial agents [35,36]. Spatial fabrication strategies based on three-dimensional printing, Janus structures, and core–shell architectures further enable the regional integration of antibacterial, antioxidant, anti-inflammatory, sensing, and tissue-regenerative modules, providing new directions for the development of multifunctional smart hydrogels [37,38].

Building on advances in antibacterial mechanisms and material/network engineering, research objectives have gradually shifted from merely “eliminating bacteria” toward “regulating the infection microenvironment and promoting tissue reconstruction”. Next-generation antibacterial hydrogels are evolving into intelligent platforms that integrate infection control, immunomodulation, oxidative stress regulation, tissue regeneration, and real-time monitoring [39,40,41,42]. Through strategies such as modulating macrophage phenotypes, scavenging excessive reactive oxygen species, promoting angiogenesis, and delivering growth factors, hydrogels can not only reduce bacterial burden in infected wounds but also alleviate excessive inflammation and oxidative stress, thereby remodeling the immune microenvironment to favor tissue repair [43,44]. The construction of stable wet-adhesive interfaces, hydrated antifouling surfaces, and low-friction surface layers can improve hydrogel fixation and adaptability on dynamic tissue surfaces, inhibit nonspecific protein deposition and initial bacterial adhesion, and reduce friction-induced damage at the material–tissue interface [45,46]. Furthermore, by integrating conductive networks, colorimetric sensors, structural colors, and enzyme-responsive modules, smart hydrogel dressings can enable real-time or visual monitoring of physicochemical signals such as wound deformation, pH, and glucose levels, thereby providing a basis for infection-risk assessment and personalized wound management [47,48]. However, most smart hydrogels currently remain at the stage of integrating diagnostic and therapeutic functions, whereas systems capable of autonomously completing the full “sensing–decision-making–intervention–feedback” cycle remain scarce [40,49]. Establishing quantitative relationships among pathological signals, bacterial burden, drug-release dosage, and therapeutic outcomes is therefore essential for achieving genuine closed-loop regulation.

Against this background, this review traces the development of antibacterial hydrogels from infection control to tissue regeneration and systematically summarizes their antibacterial mechanisms, material design strategies, and emerging directions toward intelligent systems. Following this progression, the review first introduces the major antibacterial mechanisms that provide the mechanistic foundation for pathogen elimination. It then examines material and structural construction strategies that enable these antibacterial effects to be delivered in a stable, controllable, and adaptive manner. Building on these foundations, recent advances in infection-microenvironment regulation, tissue regeneration, interface adaptation, and intelligent theranostic integration are further discussed. Finally, current challenges are summarized in terms of antibacterial evaluation systems, mechanistic understanding, precise release control, adaptability to complex environments, and long-term biosafety, followed by an outlook on the development of next-generation antibacterial hydrogels with enhanced efficacy, safety, and intelligence. Finally, the major translational hurdles from preclinical research to clinical application are discussed, including long-term biosafety, manufacturing scalability and reproducibility, sterilization compatibility and shelf-life stability.

## 2. Major Antibacterial Mechanisms of Antibacterial Hydrogels

### 2.1. Cationic Contact Killing and Polyphenol-Mediated Synergistic Mechanisms

Cationic polymers primarily interact electrostatically with negatively charged bacterial surfaces through positively charged groups, such as quaternary ammonium groups and protonated amino groups. These interactions disrupt the surface charge distribution and compromise membrane integrity, leading to increased membrane permeability, leakage of intracellular components, and bacterial inactivation. Chitosan, quaternized chitosan, and cationic polypeptides are commonly used functional components in such hydrogels. Their antibacterial efficacy mainly depends on the density of cationic groups, the degree of surface exposure, and the effective contact area between the material and bacterial cells.

**Figure 1:**
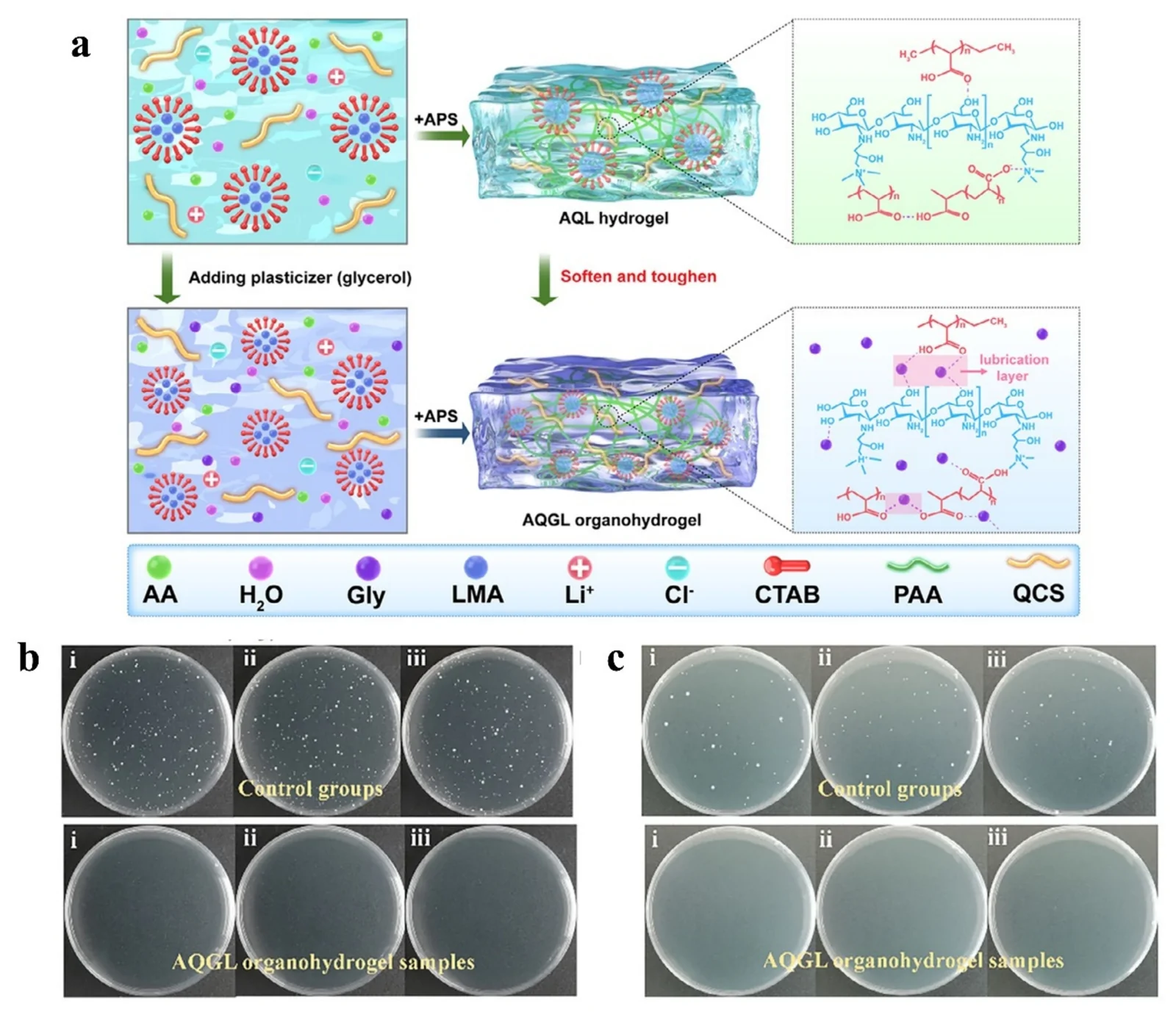
Schematic diagram of the synthesis procedure, design strategy, and chemical structure of the HAPAA/QCS/LiCl organohydrogel (**a**). Comparative antibacterial performance against *S. aureus* (**b**) and *E. coli* (**c**) for HAPAA/QCS/LiCl and control samples [50]. Reprinted with permission from Wiley−VCH GmbH of Copyright 2026.

Based on the mechanisms described above, cationic components can be integrated with different network architectures to achieve contact-mediated antibacterial activity while simultaneously improving the mechanical or conductive properties of hydrogels. In the hydrophobically associated PAA/quaternized chitosan (QCS)/LiCl (HAPAA/QCS/LiCl) organohydrogel, the quaternary ammonium groups of QCS interact electrostatically with negatively charged bacterial membranes, leading to membrane disruption and 99.9% antibacterial efficiency against both *S. aureus* and *E. coli* [50], as shown in Figure 1. The physically crosslinked PAA/Lys-BPEA hydrogel relies on cationic sites containing abundant lysine-derived amino groups to adsorb onto and disrupt bacterial membranes, thereby achieving contact-mediated killing of *E. coli* and *S. aureus* [51]. Natural cationic polymers such as bacterial cellulose–chitosan (BC/CS) similarly exert contact-killing activity through protonated amino groups that electrostatically interact with and disrupt negatively charged bacterial membranes [52]. The incorporation of polyphenols into cationic contact-killing systems can further enhance membrane damage while imparting antioxidant and adhesive properties. In chitosan/tannic acid (CS/TA) hydrogels, the cationic groups of chitosan interact electrostatically with negatively charged bacterial surfaces and disrupt cell membranes, while tannic acid further aggravates membrane shrinkage, depression, and structural damage [53].

### 2.2. Chemical Antibacterial Activity Mediated by Metal-Ion Release and Reactive Halogen Species

Metal ions such as Ag^+^, Cu^2+^, and Zn^2+^ exert antibacterial effects through multiple pathways, including disruption of bacterial membrane structures, binding to thiol-containing proteins, inhibition of key enzymatic activities, and disturbance of ionic homeostasis and energy metabolism. Some metal ions can also induce oxidative stress. Immobilizing metal ions or metallic nanoparticles within hydrogel networks can restrict their rapid diffusion and loss, reduce local cytotoxicity caused by burst release, and prolong antibacterial activity. Furthermore, regulating the release of metal species through porous carriers, dynamic coordination interactions, or stimuli-responsive structures enables sustained or on-demand antibacterial activity in response to the infection microenvironment.

Porous inorganic carriers can enhance metal-ion loading capacity while supporting both antibacterial activity and tissue repair. Metal ion-loaded hydroxyapatite composite hydrogels exploit the high specific surface area and abundant ion-binding sites of nanohydroxyapatite to achieve the localized immobilization and controlled release of Ag^+^, Zn^2+^, and Cu^2+^. Among these ions, Ag^+^ primarily damages bacterial cell walls, respiratory enzyme systems, and DNA. Zn^2+^ not only exerts antibacterial effects through membrane disruption but also regulates inflammation and promotes keratinocyte migration, collagen synthesis, and angiogenesis. Cu^2+^ induces bactericidal membrane and DNA damage through reactive oxygen species while activating vascular endothelial growth factor (VEGF) related signaling pathways to promote angiogenesis and tissue remodeling [54].

In addition to inorganic carriers, polymer networks can serve as localized reservoirs for metallic nanoparticles. Lin et al. grafted PNIPAAm onto chitosan, followed by blending and crosslinking with PVA/PVP and loading with AgNPs, to construct a thermoresponsive antibacterial hydrogel. The network functions as a localized AgNP reservoir, reducing burst release and regulating the sustained release of silver species. The released Ag^+^ exerts antibacterial effects by disrupting bacterial cell membranes, interfering with protein synthesis, and inducing oxidative stress [55]. The Ag-IMP-U@Cur composite hydrogel employs Ag^+^–IMP coordination assemblies and uridine-mediated dynamic boronate ester bonds to stabilize the network. Under acidic wound conditions, the hydrogel responsively releases uridine, curcumin, and Zn^2+^ while maintaining Ag^+^ release. Ag^+^ induces bacterial membrane disruption, binds to protein thiol groups, and triggers oxidative stress, whereas Zn^2+^ and curcumin further enhance the synergistic antibacterial effect [56].

**Figure 2:**
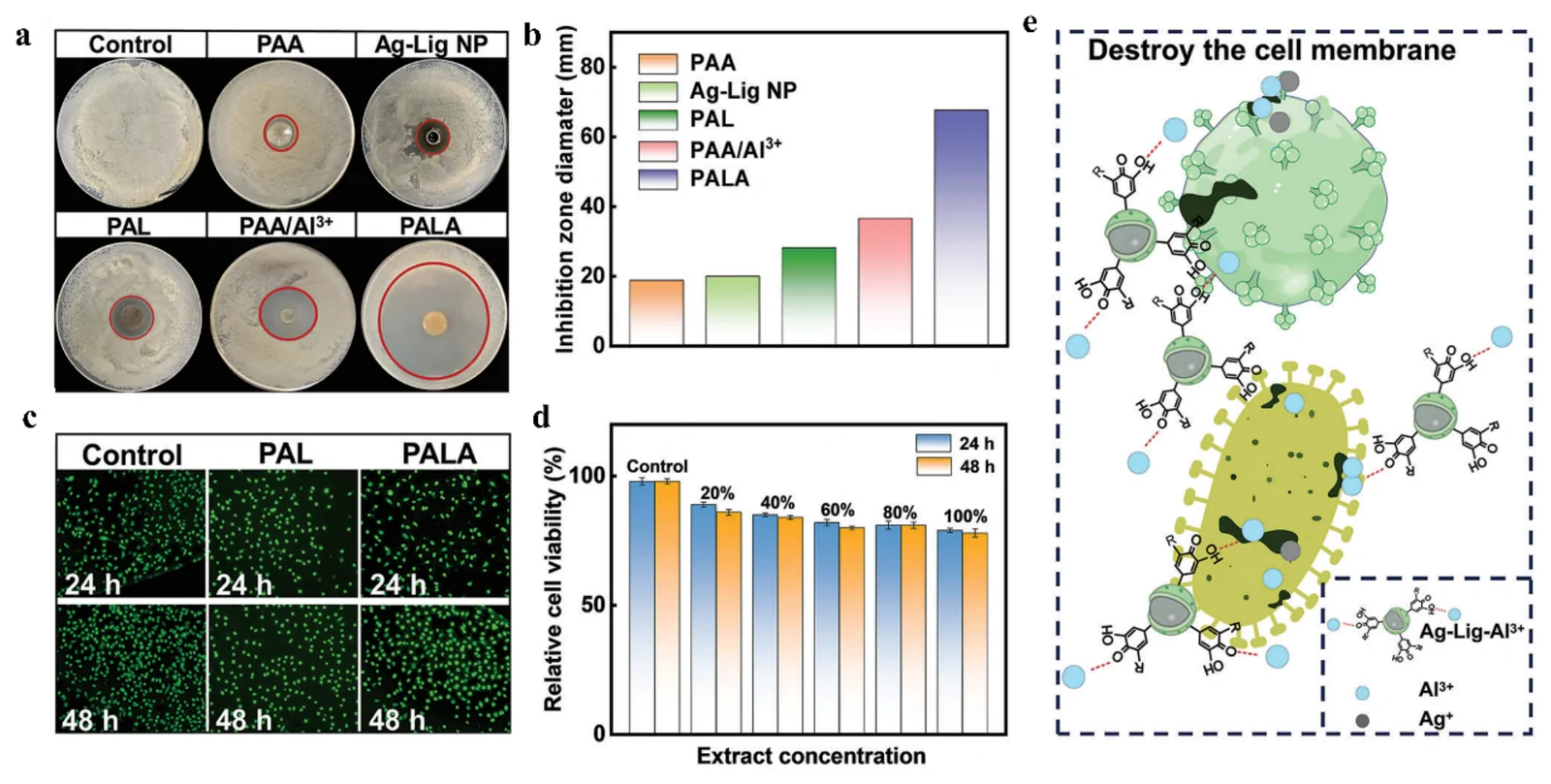
In vitro bacteriostasis and biocompatibility of hydrogel. (**a**) Antibacterial activities *(E. coli*) and (**b**) antibacterial zone diameter of PAA, Ag-Lig NP, PAL, PAA/Al^3+^, and PALA hydrogels. (**c**) Biocompatibility, and (**d**) relative cell viability of PAL, and PALA hydrogels. (**e**) Hydrogels’ antibacterial schematic diagram [58]. Reprinted with permission from Wiley−VCH GmbH of Copyright 2024.

The hierarchical architecture and crosslinking mode of hydrogels can further regulate the diffusion behavior of metal ions. Li et al. fabricated a tubular sodium alginate/carboxymethyl chitosan hydrogel dually crosslinked by Ca^2+^ and Cu^2+^ using a one-step anodic electrodeposition method. Cu^2+^ slowly diffused from the hydrogel network and exerted antibacterial effects by disrupting bacterial membranes, interfering with protein function, and inducing oxidative stress. The antibacterial efficiency decreased from 98.2% to 91.5% as the number of layers increased, indicating that interlayer barriers could retard Cu^2+^ diffusion [57]. Metal ions can also simultaneously contribute to network crosslinking, antibacterial activity, and functional integration. Yang et al. developed a PAA/Ag-Lig/Al^3+^ bimetal–phenolic hydrogel, in which Ag^+^ exerted antibacterial activity by binding to and inactivating sulfur-containing proteins in bacterial membranes, while lignin-derived polyphenols enhanced bacterial killing by disrupting the cell wall and altering membrane permeability. Phenolic hydroxyl groups, carboxyl groups, and metal-coordination sites endowed the hydrogel with strong wet-tissue adhesion. Its conductive network also enabled wound-healing monitoring through resistance changes and transmitted externally applied microcurrents to promote cell migration and tissue repair, as shown in Figure 2 [58]. Unlike antibacterial strategies that rely on metal-ion diffusion, N-halamines achieve oxidative bacterial killing primarily through the release of reactive halogen species. In the SPP (sodium alginate-Ca^2+^ (SA-Ca^2+^), polyvinyl alcohol (PVA), and chlorinated poly [2-(methacrylamido) glucopyranose] (pMAG-Cl, a typical N-halamine glycopolymer)) hydrogel, the N-halamine glycopolymer pMAG-Cl was incorporated into an SA-Ca^2+^/PVA network. Electrophilic active chlorine released from N–Cl bonds oxidized bacterial membranes and proteins. After active chlorine depletion, the inactivated hydrogel could be recharged through direct contact with a pMAG-Cl-containing hydrogel, allowing active chlorine to migrate through the highly hydrated porous channels and reform N–Cl bonds, thereby restoring antibacterial activity. This regeneration process could be repeated for at least 20 cycles, and the regenerated hydrogel maintained an *E. coli* killing efficiency close to 100% over 5 consecutive test cycles [59].

### 2.3. Nanozyme-Catalyzed Bacterial Killing and Bidirectional ROS Regulation

Infected wounds are commonly characterized by local acidification, ROS accumulation, persistent inflammation, and tissue hypoxia. Nanozyme-based hydrogels can exploit the acidic conditions and H_2_O_2_ present in the infection microenvironment to catalytically generate reactive species, such as hydroxyl radicals and singlet oxygen, which damage bacterial membranes, proteins, and nucleic acids. As the bacterial burden decreases and wound pH returns toward physiological levels, some nanozymes can shift toward scavenging excessive ROS and generating O_2_, thereby alleviating oxidative stress and tissue hypoxia. Therefore, the central objective of these systems is not simply to maximize ROS production, but rather to dynamically coordinate pro-oxidative bacterial killing with antioxidant protection according to the different stages of infection control and tissue repair.

Switching enzyme-mimetic activities in response to pH changes is a major strategy for constructing stage-specific ROS-regulating systems. The bromothymol blue (BTB)/borax/tannic acid (BT)-PVA/Dex/CaO_2_/FeS_2_ (PDCF) bilayer self-healing hydrogel integrates visual pH monitoring with a CaO_2_/FeS_2_ therapeutic layer, enabling infection-stage-dependent bidirectional ROS regulation. During the acidic phase, CaO_2_ supplies H_2_O_2_ in situ, while FeS_2_ generates ·OH, ^1^O_2_, $O_{2}^{\cdot -}$ —through its peroxidase-like activity in synergy with near-infrared photothermal effects, thereby killing bacteria and disrupting biofilms. During the neutral phase, the system shifts toward oxygen generation and catalase-like catalysis, removing residual H_2_O_2_, alleviating oxidative stress and hypoxia, and promoting tissue repair [60]. Similarly, a copper–gallic acid nanozyme/GelMA–chitooligosaccharide composite hydrogel can switch its enzyme-mimetic activities according to wound pH. Under mildly acidic conditions, it exhibits peroxidase-like activity and catalyzes H_2_O_2_ to generate ·OH for oxidative bacterial killing. As the environment approaches neutrality, it shifts to catalase-like and superoxide dismutase-like activities, decomposing H_2_O_2_, releasing O_2_, and scavenging superoxide anions. This transition alleviates oxidative stress and inflammation and enables stage-specific regulation of ROS generation and clearance [61].

**Figure 3:**
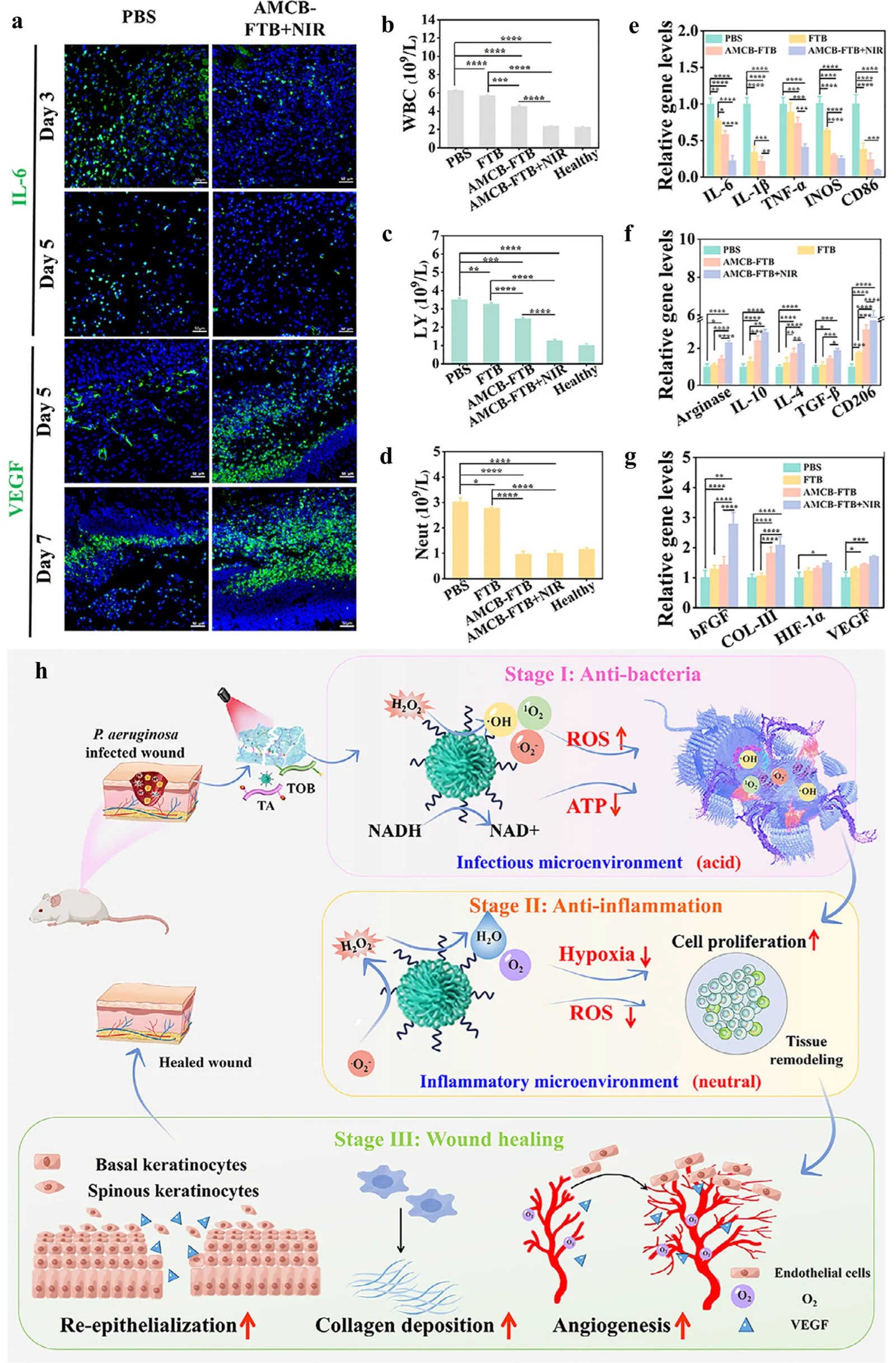
(**a**) Immunohistochemical staining of IL-6 and VEGF in the treated skin tissues. Changes in white blood cell (**b**) lymphocytes (**c**) and neutrophils (**d**) in each group after 7 days of different treatment. Data are presented as mean ± SD (n = 3). Statistical significance was tested withone-way ANOVA, * *p* < 0.05, ** *p* < 0.01, *** *p* < 0.001, **** *p* < 0.0001. (**e**) qRT-PCR results of pro-inflammatory related genes. (**f**) qRT-PCR result of anti-inflammatory-related genes. (**g**) qRT-PCR result of angiogenesis-related genes. Data are presented as mean ± SD (n = 3). Statistical significancewas tested with two-way ANOVA, * *p* < 0.05, ** *p* < 0.01, *** *p* < 0.001, **** *p* < 0.0001. (**h**) A schematic illustrates the controlled release mechanism of AMCB-FTB, highlighting its roles in antimicrobial activity, oxygen generation, oxidative stress alleviation, and tissue regeneration promotion [62]. Reprinted with permission from Wiley−VCH GmbH of Copyright 2025.

Building on this strategy, the incorporation of multiple enzyme-mimetic activities, antibacterial drugs, and photothermal effects can further improve bactericidal efficacy in complex infection environments. The AMCB-FTB smart hydrogel consists of polymyxin B-modified AuMnCu nanozymes embedded in an acid- and temperature-responsive FPBA–tobramycin–tannic acid network. Under acidic conditions, the hydrogel promotes the release of tobramycin and tannic acid, while the embedded nanozymes exert multi-enzyme-mimetic antibacterial effects by generating multiple ROS, depleting intracellular GSH, and perturbing bacterial energy metabolism through peroxidase-like, oxidase-like, NADH oxidase-like, and glutathione peroxidase-like activities (Figure 3) [62]. The H@SFMA/DMA hydrogel encapsulates an HNTs-Fe-Ag dual-enzyme nanozyme platform. In the acidic infection microenvironment, the peroxidase-like activities of Fe_3_O_4_ and Ag catalyze H_2_O_2_ to generate ·OH, while the intrinsic antibacterial activity of Ag and near-infrared photothermal effects further enhance bacterial killing. When the microenvironment becomes neutral or mildly alkaline, the system switches to catalase-like activity and decomposes H_2_O_2_ into O_2_, thereby facilitating inflammation resolution and tissue repair [63].

In addition to pH-triggered switching of enzyme-mimetic activities, metal valence-state cycling can endow nanozymes with sustained redox-regulatory capacity. CeO_2_-loaded hydrogels adapt to different microenvironments through the reversible cycling between Ce^3+^ and Ce^4+^. Under physiological or near-neutral conditions, CeO_2_ exhibits superoxide dismutase-like and catalase-like activities, scavenging $O_{2}^{\cdot -}$ and H_2_O_2_, suppressing inflammation, and promoting the migration and proliferation of fibroblasts, keratinocytes, and endothelial cells. Under acidic infection conditions, it instead exerts a localized pro-oxidative effect and assists bacterial killing through ROS-mediated membrane damage, thereby supporting both infection control and tissue regeneration [64]. Further coupling catalytic regulation with immune-microenvironment remodeling can broaden the reparative functions of nanozyme-based hydrogels. The QFP-PCC hydrogel employs a dynamic boronate ester network that responds to the acidic and ROS-rich wound microenvironment, enabling the on-demand release of Cu/Ce bimetal–phenolic nanoparticles. Cu^2+^ acts synergistically with near-infrared photothermal effects to disrupt bacterial membranes and enhance ion uptake, thereby achieving efficient bacterial killing. Meanwhile, Ce^3+^/Ce^4+^ cycling provides superoxide dismutase-like and catalase-like activities, scavenges excessive ROS, and promotes macrophage polarization from the M1 to the M2 phenotype. These effects alleviate inflammation and oxidative damage, enabling coordinated regulation of infection control and tissue repair [65].

### 2.4. Photothermally Mediated Physical Antibacterial Activity and Synergistic Sensitization

Photothermal antibacterial hydrogels are commonly loaded with photothermal agents such as indocyanine green, polydopamine, polypyrrole, MoS_2_, CuS, or black phosphorus. Under near-infrared (NIR) irradiation, these materials convert light energy into heat, enabling rapid and broad-spectrum bacterial killing by disrupting bacterial membranes, inducing protein denaturation, and disturbing cellular metabolism. However, excessive temperatures may damage normal cells and newly formed tissues. Accordingly, current design strategies have shifted from simply maximizing photothermal conversion or bactericidal efficiency toward improving therapeutic selectivity through bacterial targeting, self-limiting temperature regulation, stimuli-responsive release, ROS-mediated synergistic therapy, and tissue-reparative functions.

Integrating infection recognition and precise temperature regulation into photothermal systems facilitates on-demand treatment while minimizing damage to noninfected tissues. The BGY-ICG-R hydrogel uses gelatin and glycerol to induce hydrogen-bond-mediated topological reconstruction of bacterial cellulose, forming a transparent, flexible, adhesive, and water-retentive nanofibrous network. After loading with ICG and phenol red, the hydrogel can visually identify infection through pH-dependent color changes and simultaneously generate heat and singlet oxygen under near-infrared irradiation, thereby integrating infection monitoring with synergistic photothermal and photodynamic antibacterial therapy [66]. The Fe_2_O_3_-CS@RBP/PAOC-3@BA composite hydrogel exploits the targeting affinity of RBP toward *S. aureus* to enhance localized photothermal selectivity. When the temperature reaches approximately 47 °C, phase-transition-induced contraction forms a light-scattering barrier that limits further heating while simultaneously promoting wound-edge closure and baicalin release, thereby coupling targeted bacterial killing with inflammation alleviation and tissue repair [67]. Photothermal effects can also serve as remotely controlled triggers for the release of drugs, growth factors, and extracellular vesicles. In the VCNB-ePRP hydrogel, NIR-responsive polydopamine-containing nanobottles trigger thrombin release and activate platelet-rich plasma in situ, enabling sustained delivery of VEGF and PDGF. Together with ROS scavenging by polydopamine and vancomycin-assisted mild photothermal therapy, the system integrates methicillin-resistant *S. aureus* (MRSA) control with regenerative repair [68]. Similarly, BP/GA/GelMA-exo hydrogel microparticles exploit the photothermal effect of black phosphorus to induce a gel-to-sol transition in the glycyrrhizic acid network, thereby accelerating the on-demand release of glycyrrhizic acid and bone marrow mesenchymal stem cell-derived exosomes. The system showed strong antibacterial activity against both *E. coli* and *S. aureus* and achieved approximately 87% wound closure by day 11 in an infected diabetic wound model, supporting the integration of antibacterial, anti-inflammatory, and regenerative functions [69].

Combining photothermal effects with ROS generation or antibacterial drug release can enhance bacterial killing at relatively mild temperatures. The PEDOT@PBT-PSBMA/SF hydrogel uses an NIR-induced temperature gradient to drive thermoelectric charge separation in Bi_2_Te_3_, promoting H_2_O_2_ and singlet-oxygen generation that synergizes with localized heating to damage bacterial membranes [70]. The OBC/QAC/Ber/PPy composite hydrogel uses PPy to generate localized heat and promote the release and diffusion of berberine. Together with the membrane-disruptive activity of quaternary ammonium compounds, the system integrates photothermal damage, contact-mediated bacterial killing, and drug-based antibacterial activity into a synergistic multimodal mechanism [71]. Beyond improving bactericidal efficacy, photothermal functionality can also be integrated with tissue adhesion and antifouling interfaces. The cellulose/Poly(AM-co-MPC)/tannic acid–Fe^3+^ composite hydrogel employs a metal–polyphenol coordination network that simultaneously provides dynamic crosslinking and photothermal conversion. Combined with the intrinsic antibacterial activity of tannic acid and the antifouling properties of MPC segments, the hydrogel achieved bactericidal efficiencies of approximately 99.9% against both *S. aureus* and *E. coli* [72]. Similarly, the injectable conductive CPPFe@TA hydrogel utilizes a TA–Fe^3+^ coordination network to generate photothermal effects, while tannic acid provides additional antibacterial activity, resulting in approximately 99% inactivation of both bacterial species. Its injectability and wet-tissue adhesion further enable effective coverage of irregular wounds and improve interfacial adaptation between the material and infected tissue [73].

These antibacterial mechanisms define how bacterial killing is achieved, whereas their therapeutic effectiveness depends strongly on how the corresponding functional components are incorporated, stabilized, and regulated within hydrogel networks. The following section therefore focuses on the material and structural design principles that enable these mechanisms to operate in a controllable and clinically relevant manner.

## 3. Material Foundations and Construction Strategies of Antibacterial Hydrogels

Whereas Section 2 focuses on the biological and physicochemical mechanisms responsible for bacterial killing, this section emphasizes how hydrogel composition, network architecture, and fabrication strategies regulate mechanical stability, mass transport, functional-component retention, stimuli responsiveness, and tissue-interface adaptation. To avoid repetition, antibacterial mechanisms already discussed in Section 2 are not reiterated here unless they are directly affected by structural design.

### 3.1. Natural Polymer Scaffolds and Multilevel Structural Reinforcement

Natural polymers offer favorable biocompatibility, renewability, and abundant surface functional groups. However, hydrogels composed of a single natural polymer network often suffer from insufficient mechanical strength, limited wet-state stability, and the loss of functional components. Constructing continuous fibrous scaffolds, regulating fiber orientation and crystallization behavior, and introducing rigid–flexible coupling and hierarchical pore structures can reduce network defects and improve stress transfer and energy dissipation. These structural strategies also provide stable carriers for antibacterial agents, conductive materials, and other functional components [74].

**Figure 4:**
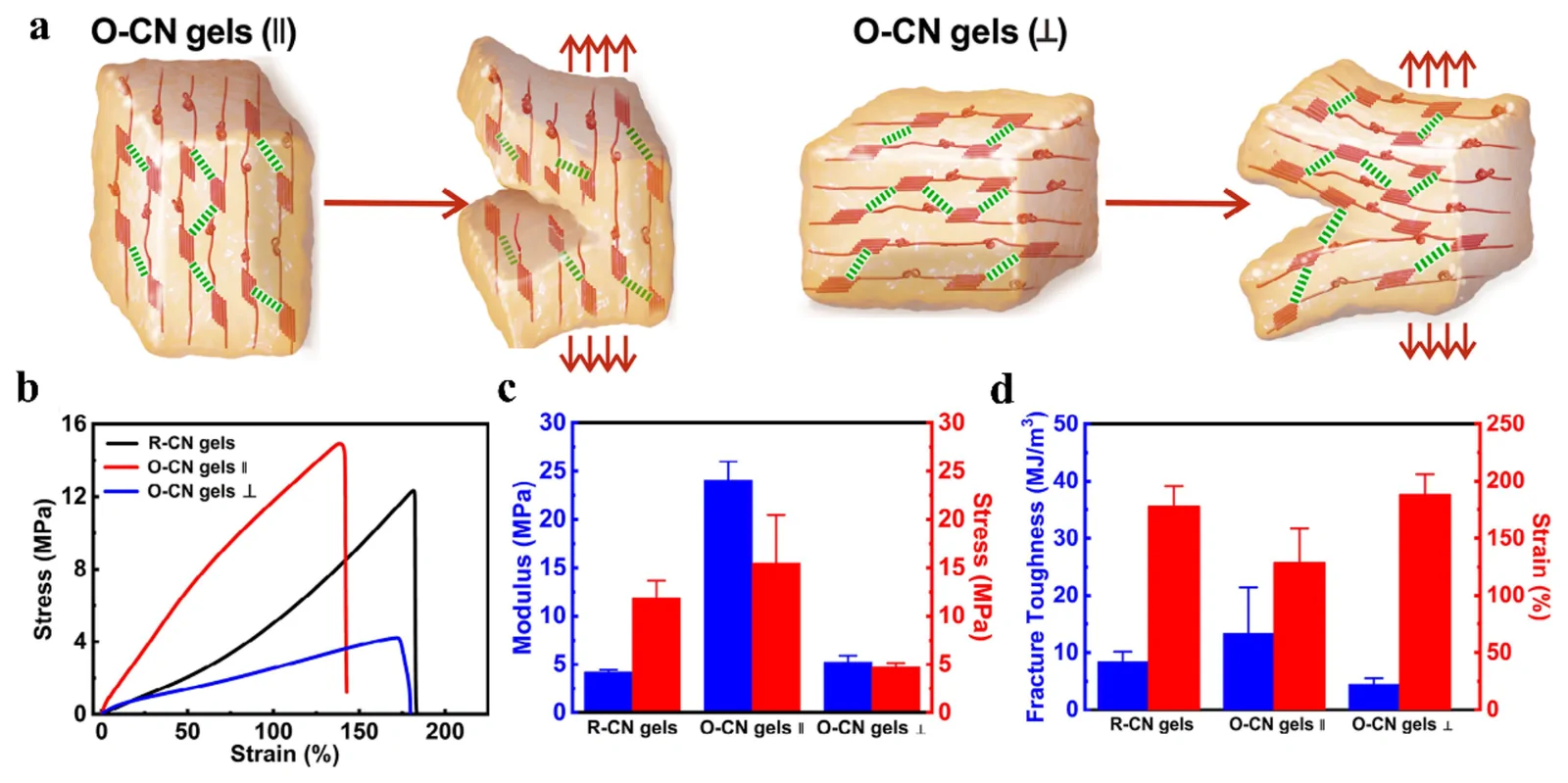
Mechanical properties of the O-CN gels after mechanical training. (**a**) Tensile test on the O-CN gels through parallel to the aligned nanofibers (║: represents the direction of cycle load parallel to the aligned nanofibers) and perpendicular to the aligned nanofibers (┴: represents the cycle load perpendicular to the aligned nanofibers) directions. (**b**) The curve of tensile stress versus tensile strain of R-CN gels, O-CN gels (║), and O-CN gels (┴). (**c**,**d**) Modulus, stress (**c**) and fracture energy, strain (**d**) of the R-CN gels, O-CN gels (║), and O-CN gels (┴) [76]. Reprinted with permission from Wiley−VCH GmbH of Copyright 2025.

Continuous nanofibrous networks provide a fundamental strategy for improving the structural stability of natural polymer hydrogels. Bacterial cellulose hydrogels exploit their high crystallinity and continuous nanofibrous networks to form natural scaffolds with few structural defects and excellent mechanical properties. The tensile strength of the nanofibers can reach approximately 2.5 GPa, while about 95% of their intrinsic stiffness is retained within the hydrogel network. Following the incorporation of functional components such as silver nanoparticles, iron oxide nanoparticles, and PEDOT:PSS, the hydrogels can integrate antibacterial activity, magnetic responsiveness, and electrical conductivity. Such continuous nanofibrous scaffolds also provide stable matrices for functional loading; for example, silver-functionalized bacterial cellulose maintained antibacterial activity for approximately 72 h [75]. Building on a continuous fibrous scaffold, regulation of fiber orientation and crystalline-domain rearrangement can further enhance the anisotropic mechanical properties of the material. The crystal-mediated aligned chitosan nanofiber hydrogel employs a sacrificial protein template to induce chitosan-chain assembly, while mechanical training promotes nanofiber alignment, crystalline-domain reconstruction, and reinforcement of interchain hydrogen bonding, thereby producing a fibrocartilage-like anisotropic architecture, as shown in Figure 4. At a water content of approximately 74 wt%, the hydrogel exhibited a tensile strength of 15.4 MPa and a Young’s modulus of 24.1 MPa. The aligned chitosan nanofiber hydrogel retained the intrinsic cationic antibacterial activity of chitosan discussed in Section 2.1, showing bactericidal efficiencies of approximately 63% against *S. aureus* and 95% against *E. coli* after 5 h [76].

In addition to artificially induced fiber alignment, the inherent hierarchical structures of natural materials can be directly employed as rigid reinforcing scaffolds. The hierarchical channels and aligned cellulose fibers of natural wood can simultaneously provide mechanical reinforcement and directional ion transport. Zhang et al. removed lignin and most of the hemicellulose from wood, infiltrated the resulting porous scaffold with a precursor solution containing lipoic acid, methacrylated gelatin, and an ionic liquid, and subsequently performed in situ polymerization. Through rigid-scaffold–flexible-network coupling and interfacial molecular interactions, the composite hydrogel achieved longitudinal and transverse tensile strengths of 327 and 68 kPa, respectively, and exhibited anisotropic electrical conductivity. In addition to its anisotropic mechanical and conductive properties, the composite hydrogel retained strong antibacterial activity, reaching approximately 99% inhibition after 48 h [77]. Unlike strategies that employ preformed fibrous scaffolds, inducing polymer-chain aggregation and crystallization enables the in situ formation of high-density physical crosslinking domains within the network. In the sodium lignosulfonate-induced PVA supramolecular hydrogel, the diffusion of sodium lignosulfonate promoted PVA-chain aggregation and the continuous formation and densification of crystalline domains. The resulting physically crosslinked network exhibited a tensile strength of approximately 20 MPa, a Young’s modulus of 14 MPa, a toughness of 50 MJ·m^−3^, and an elongation at break of approximately 500%. The hydrogel retained its tensile performance at −60 °C and additionally provided nearly 100% ultraviolet shielding together with antibacterial activity against *E. coli* and *S. aureus* [78].

### 3.2. Multiple Crosslinking, Dynamic Coordination, and Self-Healing Networks

A single crosslinked network generally cannot simultaneously satisfy the requirements of antibacterial hydrogels for mechanical strength, toughness, adhesion, and damage-recovery capability. Multiple-crosslinking strategies couple stable structural frameworks with dynamic reversible interactions. Permanent covalent networks, crystalline domains, or continuous physical networks maintain the overall morphology and facilitate load transfer, whereas hydrogen bonds, ionic bonds, metal–ligand coordination bonds, and dynamic covalent bonds dissipate energy through reversible dissociation and reformation, thereby promoting network self-healing [79,80,81,82,83]. The synergy among different crosslinking mechanisms can further improve the wet-state stability, interfacial adaptability, and functional-component immobilization capacity of hydrogels, as summarized in Table 1.

Therefore, the central principle of multiple-crosslinking design lies in the functional division between stable scaffolds and dynamic sacrificial bonds, rather than simply increasing the crosslinking density. Stable networks are responsible for maintaining structural integrity and transmitting loads, whereas reversible crosslinks enable energy dissipation, self-healing, and adaptation to tissue interfaces through dynamic bond exchange. Metal ions, polyphenols, and cationic components that simultaneously provide crosslinking and antibacterial functions can further promote the integration of structural and therapeutic properties.

### 3.3. Environmental Adaptability and Endogenous/Exogenous Stimuli-Responsive Release

The structure and functionality of antibacterial hydrogels are susceptible to low temperatures, dehydration, and complex pathological microenvironments, potentially resulting in freezing-induced embrittlement, decreased electrical conductivity, interfacial instability, or unintended release of active components. Regulating the binding state of water molecules, incorporating cryoprotective solvents, and maintaining stable hydration layers can improve material stability under low-temperature and dynamic wet conditions. Furthermore, endogenous pathological signals, such as glucose, pH, and reactive oxygen species, as well as exogenous stimuli such as near-infrared light, can be used to regulate network swelling, degradation, and molecular diffusion, thereby enabling the sustained or on-demand release of antibacterial and tissue-reparative components.

Regulating the state of water molecules is a major strategy for improving the low-temperature stability of hydrogels. A multicomponent conductive organohydrogel was constructed with a dynamic supramolecular network comprising silk fibroin nanofibers, catechol-grafted chitosan, tannic acid, and Mg^2+^/Ca^2+^, while an ethylene glycol/water binary solvent was used to reconstruct the hydrogen-bonding network of water molecules and inhibit crystallization at low temperatures. The hydrogel remained flexible at −18 °C, with its electrical conductivity decreasing only from 1.76 mS·cm^−1^ at 25 °C to 1.43 mS·cm^−1^. Through the synergistic effects of chitosan, tannic acid, and metal ions, it achieved inhibition rates of over 95% against *S. aureus* and over 99% against *E. coli*. Its ABTS radical-scavenging rate reached approximately 99%, and it accelerated diabetic wound healing by suppressing inflammation and promoting angiogenesis [90]. Similarly, a multiple-crosslinked ionically conductive PVA/sodium alginate/phytic acid/CaCl_2_/glycerol hydrogel was constructed through hydrogen bonding, Ca^2+^ coordination, and alginate ionic crosslinking. Glycerol replacement of free water, together with the synergistic regulation of water molecules by phytic acid and inorganic ions, preserved low-temperature flexibility and ionic transport. The hydrogel also inhibited bacterial growth through the chelation of metal ions and proteins by phytic acid, combined with the synergistic membrane-damaging effect of CaCl_2_. After 24 h of incubation, it almost completely inhibited and reduced the number of *S. aureus* by approximately 95.04% [91]. In addition to low-temperature adaptability, maintaining a stable hydrated interface can improve the durability of hydrogels in dynamic wet environments. A gradient microporous–hydrated polymer brush photothermal composite hydrogel coating employed a dense network on the substrate side to provide interfacial bonding and mechanical support. Meanwhile, surface micropores served as water reservoirs, and sulfonated polymer brushes within the pores formed a stable hydration layer, reducing the coefficient of friction in water to approximately 0.04. Under 808 nm near-infrared irradiation, MoS_2_ nanosheets incorporated into the coating generated localized photothermal effects, achieving bactericidal efficiencies exceeding 99% against both *E. coli* and *S. aureus* within 10 min. Thus, the coating combined a low-friction interface with efficient photothermal antibacterial activity [92].

Controlled release can also be achieved through carrier-mediated compartmentalization without relying on complex stimuli-responsive networks. For example, alginate/tetracycline beads embedded within a chitosan/pluronic/agarose hydrogel prolonged tetracycline release compared with direct drug loading, while maintaining antibacterial activity against Gram-positive and Gram-negative bacteria and favorable compatibility with HFF2 cells [93]. With respect to endogenous stimuli responsiveness, elevated glucose levels and local acidification in wounds can be converted into signals for controlled therapeutic release. A glucose-responsive, exosome-loaded self-healing hydrogel was constructed through reversible Schiff-base bonds between the aldehyde groups of oxidized pullulan and the amino groups of carboxymethyl chitosan. Grafted glucose oxidase (GOx) converted excess glucose into gluconic acid and H_2_O_2_. The resulting local acidification promoted network degradation and exosome release, thereby establishing a dual glucose/pH-responsive controlled-release mechanism. The hydrogel achieved synergistic antibacterial activity through glucose depletion, H_2_O_2_ generation, and carboxymethyl chitosan-mediated bacterial membrane disruption [94].

Combining endogenous pH signals with exogenous near-infrared irradiation can further improve the controllability of therapeutic release. pH/NIR dual-responsive tea polyphenol-loaded carboxymethyl chitosan/sodium alginate/MXene hydrogel microspheres were constructed through Ca^2+^ crosslinking, while carboxyl-group ionization regulated network swelling and erosion. At pH 1.8, less than 13% of the tea polyphenols were released within 24 h, whereas the cumulative release increased to approximately 50% and 65% at pH 6.8 and 7.8, respectively. Upon near-infrared irradiation, the photothermal effect of MXene further increased the cumulative release at 6 h by approximately 9%. This system therefore protected the payload under acidic conditions while enabling accelerated release under near-neutral, mildly alkaline, and light-stimulated conditions, together with antioxidant and antibacterial activities [95]. Similarly, a pH/NIR dual-responsive metformin-loaded hyaluronic acid hydrogel was constructed through dynamic imine bonds and Fe^3+^–polyphenol coordination, forming a reversible network. Under acidic wound conditions, the two dynamic interactions dissociated synergistically and accelerated hydrogel degradation, enabling on-demand metformin release. At pH 5.5, the cumulative release reached 87.9% within 3 h, whereas sustained release was observed at pH 7.4. The Fe^3+^–polyphenol coordination structure also generated heat in response to near-infrared irradiation, achieving a photothermal bactericidal efficiency exceeding 99.9% [96]. More advanced multistimuli-responsive smart hydrogels can simultaneously sense wound pH, ROS, electrical signals, and mechanical changes. pH-responsive mechanisms regulate the release of active components, ROS-responsive structures alleviate oxidative stress, and conductive networks promote cell migration, angiogenesis, and tissue remodeling. Their dynamic mechanical networks can also improve conformability, sealing, and contraction on irregular wounds, thereby enabling multidimensional regulation of the wound microenvironment and tissue-repair process [97].

### 3.4. Advanced Manufacturing and Spatial-Structure Engineering

Advanced manufacturing and spatial-structure engineering can improve the structural controllability of antibacterial hydrogels at three levels: external geometry, internal pore architecture, and functional compartmentalization. Three-dimensional printing enables the fabrication of personalized geometries tailored to wound morphology. Interconnected porous networks facilitate exudate drainage, oxygen and nutrient exchange, and cell migration. Janus and core–shell structures allow antibacterial, antioxidant, and tissue-reparative components to be spatially distributed in distinct regions, thereby minimizing interference among functional components and enabling directional transport and spatiotemporally coordinated therapy [98].

Three-dimensional printing provides an effective approach for the personalized shaping and multifunctional integration of antibacterial hydrogels. The PVA/SA/NaCl/PHMB antibacterial sensing hydrogel forms a printable network through freeze–thaw-induced physical crosslinking and hydrogen bonding between PVA and SA. At an SA content of 1.2%, the hydrogel exhibited a fracture stress of 226 kPa and a fracture strain of 325%. Its gauge factors were 1.89 and 3.29 over the strain ranges of 0–100% and 100–240%, respectively, with response and recovery times of approximately 195 and 198 ms. These properties enable the monitoring of body movements, vocalization, and electromyographic signals. Through electrostatic adsorption and membrane disruption, PHMB endowed the hydrogel with stable antibacterial activity against *E. coli* and *B. subtilis* for up to 7 days [99]. Similarly, the P407/QCS-PAAm hydrogel forms a stable network through MBAA-mediated covalent crosslinking, hydrogen bonding, and chain entanglement. P407 imparts a tunable thermoresponsive sol–gel transition to the precursor solution, allowing complex structures to be fabricated through extrusion printing followed by ultraviolet curing. QCS simultaneously provides an ionic conductivity of 0.29 S·m^−1^ and antibacterial activity, achieving killing efficiencies of 93.08% against *E. coli* and 72.09% against *B. subtilis*. The printed compressive sensor exhibited stable cyclic responses and could be used for gait monitoring, thereby integrating antibacterial activity, sensing capability, and spatially controlled architecture within a single system [100].

Beyond macroscopic shape customization, hierarchical pore architectures can markedly improve mass transport and tissue ingrowth within hydrogels. The MXene-induced sponge-like hierarchical macroporous SMPAAM hydrogel was fabricated by catalyzing the rapid decomposition of APS while simultaneously immobilizing gas bubbles, thereby generating interconnected macropores of 200–300 μm and secondary pores of 10–50 μm on the pore walls. This structure increased water and blood transport rates by more than 20-fold and provided a spatial foundation for nutrient exchange, cell migration, and vascular ingrowth. The hydrogel also exhibited swelling resistance, TiO_2_@C-mediated visible-light antibacterial activity, and MADA/MXene-mediated synergistic antioxidant properties. It achieved inhibition rates of approximately 86% against *E. coli* and 75% against *S. epidermidis*, together with a DPPH radical-scavenging rate of 96%, and increased the 7-day closure rate of diabetic wounds to approximately 81% [101]. Further spatial compartmentalization of functions can prevent mutually antagonistic processes, such as pro-oxidative bacterial killing and antioxidant protection, from occurring within the same region. The NiCo_2_O_4_/ZnO bilayer Janus hydrogel consists of a dense PAM outer layer loaded with NiCo_2_O_4_ nanozymes and a porous GelMA-PBA@ZnO/HA-DA inner layer containing ZnO microspheres, thereby spatially separating antioxidant and antibacterial functions. Under acidic and H_2_O_2_-rich conditions, the hydrogel accelerates Zn^2+^ release and synergistically disrupts bacteria and inhibits biofilm formation through ionic toxicity, ROS generation, and contact-mediated damage [102]. Core–shell and directionally aligned channel structures can also spatially separate different material- and energy-transport processes. For example, a tree-trunk-inspired core–shell SA/AF/CuS hydrogel employed aramid fiber-supported vertical sodium alginate channels to facilitate capillary water transport and structural reinforcement, while a dense CuS surface layer enhanced photothermal conversion and reduced the evaporation enthalpy of interfacial water, thereby spatially separating water transport from evaporation. Cu^2+^ within the hydrogel network additionally disrupted bacterial membranes and interfered with intracellular proteins and enzymatic activities, resulting in antibacterial efficiencies of 99.97% against *E. coli* and 99.92% against *S. aureus* [103]. However, because this material was primarily developed for water evaporation and antimicrobial water treatment, its direct applicability to wound therapy requires further validation.

## 4. Intelligent Responsiveness, Multifunctional Synergy, and Theranostic Integration

### 4.1. Regulation of the Infection Microenvironment and Sequential Tissue Repair

The healing of infected wounds depends not only on bacterial eradication but also on the simultaneous alleviation of oxidative stress and persistent inflammation, as well as the promotion of angiogenesis, collagen deposition, and tissue reconstruction [104]. Accordingly, multifunctional hydrogels increasingly integrate antibacterial activity, ROS regulation, immunomodulation, and regenerative factor delivery within a single platform. By sensing wound-associated signals such as pH, ROS levels, and temperature, these systems can coordinate infection control with tissue repair. Because therapeutic requirements vary across different stages of wound healing, the design focus has shifted from the simple combination of multiple functions toward their stage-specific and sequential coordination [105].

Pathological signal-triggered release of immunomodulatory factors can integrate oxidative stress regulation with reparative immune remodeling. A ROS-responsive IL-4-loaded CMCS/β-CD/Fc supramolecular hydrogel was constructed through β-CD/Fc host–guest interactions to form a dynamic network. In an H_2_O_2_-rich environment, Fc is oxidized, weakening the host–guest inclusion interactions and promoting network dissociation and on-demand IL-4 release. Meanwhile, Fc consumes H_2_O_2_, and CMCS scavenges the subsequently generated ·OH, thereby regulating oxidative stress. The released IL-4 promotes macrophage polarization from the M1 to the M2 phenotype, enabling a gradual therapeutic transition from early-stage inflammation suppression to angiogenesis and tissue repair. The CMCS/Fc components also inhibit *S. aureus* and *E. coli* by disrupting bacterial membrane integrity [106]. Building on this strategy, combining platelet-derived growth factor delivery with ROS responsiveness can further promote vascular reconstruction. A ROS-responsive PRP-loaded double-network hydrogel integrated CaTA and PRP through dynamic boronate ester bonds and a photocrosslinked MA-CMCS network. Elevated ROS levels triggered the dissociation of boronate ester bonds and the on-demand release of TA and Ca^2+^. TA provided free-radical-scavenging and antibacterial activities, whereas Ca^2+^ activated PRP in situ, enabling the sustained release of VEGF, PDGF, and EGF [107]. In addition to ROS responsiveness, temperature- and pH-responsive regulation can be employed to extend the therapeutic window of active components. A temperature/pH dual-responsive NAR-FA NLC-loaded P407/CMCS hydrogel exploited P407 micellization and gelation at 37 °C, together with CMCS carboxyl-group ionization-mediated regulation of network swelling, to achieve the sustained release of NAR and FA over 48 h. The hydrogel significantly inhibited *B. subtilis* and *E. coli*. By upregulating VEGF and reducing ICAM-1 expression, it promoted the transition of wounds from the inflammatory phase to the proliferative and remodeling phases. In diabetic rats, the hydrogel achieved a wound-closure rate of 93.2 ± 3.4% on day 15, demonstrating the synergistic integration of antibacterial activity, inflammation regulation, and tissue regeneration [108].

However, infection control, inflammation resolution, and tissue regeneration do not occur simultaneously. During the early stage of wound healing, wounds are typically characterized by a high bacterial burden, acidification, ROS accumulation, and intense inflammation; therefore, hemostasis and pathogen elimination should be prioritized. As the infection subsides, the therapeutic focus shifts toward scavenging excessive ROS, restoring cellular metabolism, promoting angiogenesis, and facilitating tissue reconstruction. Simultaneous release of multiple functional components may result in mismatched therapeutic timing, premature inactivation of regenerative factors, or interference among different functions. Accordingly, sequential therapeutic hydrogels increasingly exploit disease-stage-dependent changes in pH and ROS levels to activate antibacterial, anti-inflammatory, and regenerative functions in a programmed, stage-specific manner.

Cascade responsiveness to pH and ROS can enable a progressive functional transition from infection eradication to metabolic support and tissue regeneration. The pH/ROS dual-responsive CBGCT hydrogel responds to the infection microenvironment through the cleavage of dynamic boronate ester bonds and the dissociation of Cu@TA nanosheets, resulting in the cascade release of Cu@TA, Cu^2+^, and TA. Together with the contact-killing activity of residual primary amino groups, these components synergistically enhance MRSA eradication. Subsequently, TA and poly(citric acid) components scavenge ROS and suppress inflammation, while citrate enhances the tricarboxylic acid cycle and ATP production, and Cu^2+^ activates HIF-1α/VEGF-related angiogenic signaling. These effects enable a sequential transition from infection control to metabolic support and tissue regeneration [109]. Further coupling distinct pathological signals to different release stages can improve the controllability of sequential therapy. The pH/ROS dual-responsive N-Gel/ODex-nZnO/MIC@Pf hydrogel forms an injectable and self-healing network through dynamic Schiff-base bonds, while its high fluid absorption capacity, cationic groups, and RGD sequences promote early hemostasis. An acidic environment triggers network dissociation and the rapid release of nZnO, which eliminates *S. aureus* and *P. aeruginosa* through membrane disruption, lipid oxidation, and ROS generation. Elevated ROS levels subsequently cleave thioketal-containing micelles and enable the sustained release of paeoniflorin, which upregulates HIF-α/VEGF signaling, reduces TNF-α expression, and increases IL-10 and CD31 expression. The system therefore achieves a sequential transition from hemostasis and antibacterial treatment to inflammation regulation and vascular regeneration [110]. Similar stage-specific regulation can also be extended to the repair of infected bone tissue. The thermosensitive GA-HBC-LIC hydrogel undergoes in situ gelation within periodontal pockets and maintains space for tissue regeneration. During the early stage, chitosan, gallic acid, and Zn^2+^ synergistically disrupt bacterial membranes and inhibit biofilm formation, thereby reducing the burdens of *P. gingivalis* and *S. aureus*. Subsequently, gallic acid scavenges ROS, while the sustained release of Zn^2+^ targets ZEB1 and promotes macrophage polarization from the M1 to the M2 phenotype. Icariin and Zn^2+^ further enhance the expression of ALP, RUNX2, SP7, and type I collagen, enabling a progressive transition from infection control and inflammation resolution to osteogenic regeneration [111].

### 4.2. Tissue–Interface Adaptation and Mechanical Signal Sensing

Antibacterial hydrogels used for tissue repair must simultaneously satisfy two distinct interfacial requirements: stable tissue attachment and resistance to biofouling. The tissue-contacting side should maintain wet adhesion and mechanical sealing under exudate flushing, tissue movement, and local pressure, whereas the side exposed to body fluids or the external environment should suppress protein deposition, bacterial adhesion, and biofilm formation. Accordingly, current designs commonly combine tissue adhesion mediated by catechol groups, polyphenols, or dynamic covalent bonds with zwitterionic structures, hydration layers, and lubricating interfaces, while incorporating active antibacterial components to eliminate already adhered bacteria. The further introduction of conductive networks enables tissue deformation and interfacial stress to be converted into electrical signals, thereby allowing dynamic monitoring of wound movement and healing status [112,113].

Stable wet adhesion and defect sealing are fundamental to tissue–interface regulation. A protein-responsive tannic acid-loaded gelatin/poly(acrylic acid) adhesive hydrogel forms noncovalent complexes between tannic acid and leaked digestive proteins, thereby increasing the network modulus and interfacial adhesion strength and maintaining stable sealing of porcine small-intestinal perforations in simulated intestinal fluid. Tannic acid also endows the hydrogel with potent bactericidal activity against *E. coli* and *B. subtilis*, enabling the simultaneous mechanical protection of anastomotic sites and control of postoperative infection [114]. The introduction of an antifouling hydration layer into an adhesive system can further create a dynamic interface that combines tissue fixation with resistance to bacterial adhesion. The dynamically multiple-crosslinked OHMA hydrogel forms a tough, self-healing network through reversible Schiff-base bonds, hydrogen bonding, ionic interactions, and chain entanglement, allowing it to buffer mechanical stresses caused by wound movement. Catechol and zwitterionic groups further maintain stable wet adhesion. The hydrogel establishes a durable antibacterial barrier against *S. aureus* and *E. coli* by suppressing bacterial adhesion through its hydration layer and disrupting bacterial cell walls through cationic groups. It also promotes wound closure, tissue regeneration, and collagen deposition, thereby integrating dynamic interfacial protection with tissue repair [115].

**Figure 5:**
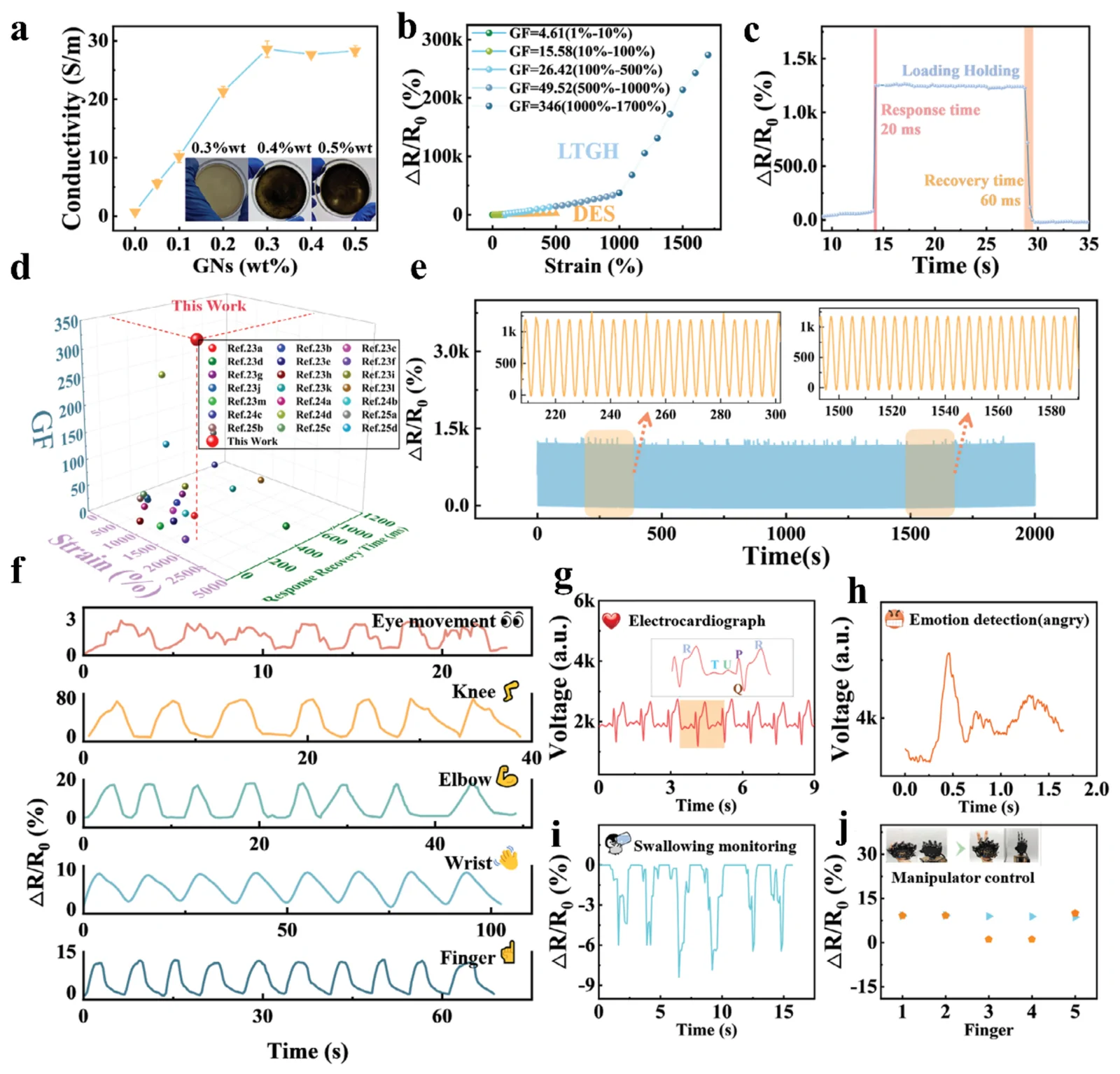
The conductive properties and sensing behaviors of LTGH: (**a**) The relationship between LTGH conductivity and GNs content (*n* = 5); (**b**) Gauge factor of LTGH at 0–1700% strain; (**c**) Response and recovery time of LTGH at 100% strain; (**d**) Comparison between LTGH and other reported strain sensors; (**e**) Electrical properties of LTGH under 1000 repetitions of 100% tensile test; (**f**) Motion detection of LTGH installed on the eye, knee, elbow, wrist and finger; (**g**) LTGH used as a physiological electrode for heart rate detection; (**h**) Emotion detection; (**i**) Swallowing detection; (**j**) Optical images and resistance changes of LTGH controlling a robotic hand, from a fist to a “Victory” gesture [118]. Reprinted with permission from Wiley−VCH GmbH of Copyright 2025.

For implantable devices such as catheters, interfacial design must also balance coating stability with low-friction performance [116]. A WPU-stabilized PNSQ hydrogel coating for catheters employs a waterborne polyurethane adhesive layer to establish strong interfacial bonding, while QCS, SBMA, and NVP form a hydration layer that reduces the coefficient of friction to 0.0357, thereby minimizing tissue damage caused by friction during device insertion. Zn^2+^ is released rapidly during the initial stage and continuously thereafter, exerting prolonged antibacterial effects against *E. coli* and *B. subtilis* by disrupting bacterial membranes and interfering with nucleic acid and enzymatic functions. This design therefore integrates interfacial stability, low friction, and sustained antibacterial protection [117]. After stable tissue attachment has been achieved, conductive networks can further convert interfacial deformation into detectable signals. A graphene-based conductive antibacterial hydrogel stabilized by lignin–tannin nanospheres suppresses graphene-sheet aggregation by anchoring the sheet edges and defect sites, thereby forming a homogeneous conductive network with an electrical conductivity of 28 S·m^−1^. The hydrogel also exhibits high stretchability and a tissue adhesion strength of approximately 51.3 kPa. It converts interfacial deformation into stable resistance signals for monitoring human movement and physiological activity. Graphene-mediated charge transfer or ROS generation additionally disrupts bacterial membranes, resulting in antibacterial efficiencies of 93.6% against *E. coli* and 94.7% against *S. aureus*. Thus, the hydrogel integrates tissue attachment, mechanical sensing, and infection control, as shown in Figure 5 [118]. Similarly, the PDA-CNTs/CMC/PAA semi-interpenetrating conductive antibacterial hydrogel forms a multiple-network structure through MBA-mediated crosslinking, CMC-chain interpenetration, and dynamic Al^3+^ coordination. It exhibits an elongation at break of 1904%, a toughness of 1109.8 kJ·m^−3^, and stable adhesion to porcine skin. PDA coating promotes the homogeneous dispersion of CNTs and, together with Al^3+^, establishes synergistic electronic–ionic conduction pathways, enabling the hydrogel to convert tensile and compressive deformation into resistance signals. PDA-CNTs also inhibit *S. aureus* and *E. coli* through ROS generation, thereby integrating interfacial adhesion, mechanical sensing, and antibacterial activity. However, the antibacterial evidence was derived primarily from in vitro agar diffusion assays, and its applicability to wound treatment remains to be validated in vivo [119].

### 4.3. Theranostic Integration and Quasi-Closed-Loop Regulation

Smart antibacterial hydrogels can detect wound deformation, temperature, pH, glucose levels, and bacteria-associated signals, and subsequently deliver treatment through photothermal therapy, drug release, or metal ion-mediated antibacterial mechanisms. Theranostic designs integrate wound-state recognition and therapeutic modules within a single platform, whereas true closed-loop regulation further requires the detected signals to directly trigger and dynamically adjust treatment intensity and duration. At present, most systems remain at the stage of functional integration or manually controlled quasi-closed-loop operation. Their practical application is still limited by insufficient signal specificity, susceptibility to environmental interference, and incompatibility between diagnostic and therapeutic modules. For example, the PAI/NS–TGL–PAI/NS sandwich patch enables real-time monitoring of patch deformation and skin conformability through resistance changes, while temperature-triggered color changes provide visual feedback on the local thermal state. Therapeutically, the system integrates near-infrared photothermal therapy with I_2_-releasing antibacterial activity, thereby combining deformation and temperature monitoring with dual-mode antibacterial treatment [120]. However, the monitoring signals primarily serve as indicators of the local state and cannot directly regulate photothermal intensity or antibacterial agent release. Therefore, the system essentially represents the functional integration of monitoring and therapy rather than a fully autonomous feedback-controlled closed-loop platform. Similarly, a dressing composed of an Ag/AgCl antibacterial layer and an enzyme-responsive colorimetric layer exploits bacterial β-galactosidase to trigger a yellow-to-red color transition, enabling visual detection of bacteria-associated enzymatic activity, while Ag/AgCl-derived silver ions provide antibacterial effects [121]. Although this design integrates infection detection and antibacterial treatment within a single dressing, its detection capability is largely restricted to lacZ-positive bacteria, thereby limiting its applicable bacterial spectrum. More importantly, the diagnostic signal does not feed back to regulate silver-ion release, indicating that direct functional coupling between the diagnostic and therapeutic modules remains lacking.

Stimuli-responsive smart hydrogels further integrate environmental sensing with drug release and photothermal therapy. For example, the PNiPAAm/graphene inverse-opal hydrogel–SLIPS system exhibits a temperature-induced structural color transition from red to blue, enabling visual monitoring of local thermal changes. It combines graphene-mediated photothermal therapy, imipenem release, and the anti-adhesive properties of the SLIPS interface, thereby integrating structural-color sensing, photothermal antibacterial treatment, antibiotic delivery, and antifouling functions within a single platform [122]. Although this system demonstrates a relatively high degree of multifunctional integration, both its structural-color and temperature responses may be susceptible to environmental interference, while the drug-release parameters and long-term interfacial stability remain to be further optimized. Therefore, although the responsive signals can reflect changes in the local microenvironment, they are not yet sufficiently robust or quantitative to serve as reliable therapeutic decision-making cues. In addition, spatial compartmentalization provides another strategy for multiparametric wound monitoring. A spatially compartmentalized PVA multisensor hydrogel bandage can monitor pH and glucose through colorimetric or fluorescent signals, while resistance changes are used to detect wound- or dressing-associated deformation. The system further incorporates the near-infrared photothermal effect of PVA–CNT materials for on-demand antibacterial treatment [47]. Compared with single-parameter sensing systems, this strategy enables the simultaneous acquisition of multiple wound-microenvironment-related signals and integrates multiparametric monitoring with photothermal therapy within a single platform, showing greater potential for theranostic integration. However, its resistance to signal interference remains insufficient, biosafety evaluation is still incomplete, and validation in animal models is limited. These shortcomings indicate that substantial challenges remain before such systems can achieve stable, long-term performance under complex clinical conditions.

Overall, existing theranostic antibacterial hydrogels can monitor wound status through resistance changes, colorimetric signals, structural colors, fluorescence, and enzyme-responsive signals, while integrating these sensing functions with photothermal therapy, metal ion-mediated antibacterial activity, or drug release. Layered architectures and spatial functional compartmentalization can reduce interference between sensing and therapeutic modules. However, most systems remain at the stage of functional coexistence or quasi-closed-loop operation following a “detection–manual assessment–external activation” process. Although some stimuli-responsive networks can directly use pH, ROS, or bacteria-associated enzymes to trigger material degradation and the release of therapeutic components, they are not yet capable of autonomously adjusting treatment dosage and duration according to dynamic changes in pathological signals and therapeutic outcomes. Beyond these functional limitations, the translation of advanced antibacterial hydrogels into clinically applicable products is further constrained by long-term biosafety, manufacturing reproducibility, sterilization compatibility, storage stability, and regulatory requirements, as discussed below.

### 4.4. Translational Hurdles from Preclinical Research to Clinical Application

At present, most antibacterial hydrogels are still evaluated primarily using in vitro monoculture systems and rodent wound models to assess their therapeutic efficacy and safety. However, these simplified models differ substantially from the complex pathological environment of real chronic wounds. Clinically, chronic wounds are characterized by dynamic and highly heterogeneous microbial communities, often accompanied by polymicrobial colonization, biofilm formation, persistent inflammation, tissue hypoxia, and underlying host diseases. Therefore, commonly used single-species infection models based on *S. aureus* or *E. coli* cannot adequately reproduce either microbe–microbe interactions or microbe–host interactions [123]. In particular, biofilm formation markedly increases bacterial tolerance to antimicrobial treatment, while its spatial heterogeneity and polymicrobial composition further limit the ability of planktonic bacterial killing assays to predict the actual anti-infective efficacy of hydrogels in wounds. Future studies should therefore progressively incorporate polymicrobial biofilm models, clinically derived bacterial strains, and evaluation systems that more closely mimic the microenvironment of chronic wounds. In addition, substantial differences exist between animal models and human wounds in terms of skin architecture, immune responses, vascular status, and mechanisms of wound closure. For example, wound closure in rodents relies to a considerable extent on skin contraction, whereas human wound healing depends more heavily on re-epithelialization and granulation tissue formation. Consequently, the excellent performance of some materials in small-animal models may not translate directly into clinical efficacy [124,125,126]. To facilitate cross-system evaluation beyond individual antibacterial mechanisms, Table 2 compares representative hydrogel systems in terms of antibacterial performance, biocompatibility, and major therapeutic trade-offs. In addition to model relevance, long-term biosafety represents another major barrier to the clinical translation of complex antibacterial hydrogels. For systems containing metallic components such as Ag, Cu, and Zn, the local release dose, prolonged exposure, and potential tissue or systemic accumulation require further clarification. Even for clinically used silver-containing dressings, consistent and sufficient evidence linking in vivo silver-ion release levels to cytotoxicity remains lacking [127]. Similarly, the biological behavior of nanozymes and conductive nanofillers such as graphene and carbon nanotubes is influenced by multiple factors, including particle size, composition, surface chemistry, degradability, and dose. Their long-term tissue retention, degradation, metabolic clearance, and potential inflammatory or oxidative stress effects have not yet been fully elucidated [128,129].

Excellent performance of antibacterial hydrogels at the laboratory scale does not necessarily imply that the same material system can be directly translated into stable and reproducible large-scale manufacturing. Many hydrogels can achieve well-defined network structures at the milliliter or gram scale through precise control of raw-material ratios, mixing sequence, reaction time, and environmental conditions. However, upon scale-up, mixing efficiency, mass transfer, crosslinking kinetics, and the dispersion of functional components may all change, resulting in deviations in gelation time, mechanical properties, swelling behavior, and drug-release profiles from those observed at the laboratory scale [130,131]. Such reproducibility issues may first arise from batch-to-batch variations in the raw materials themselves. This is particularly relevant for natural or semi-synthetic polymers such as chitosan, hyaluronic acid, gelatin, and alginate, for which subtle variations in molecular weight and its distribution, purity, and degree of substitution or functionalization can further alter crosslinking density, gelation kinetics, and the resulting network properties [130]. For composite hydrogels containing nanoparticles or drug payloads, particle size and dispersion uniformity, aggregation state, drug loading, and encapsulation efficiency must likewise remain consistent during scale-up, because variations in processing parameters such as mixing, homogenization, or ultrasonication may lead to batch-to-batch fluctuations in release behavior and antibacterial performance [131,132]. As hydrogel design evolves from homogeneous networks toward spatially programmed architectures such as Janus, core–shell, multilayer, and 3D-printed structures, achieving manufacturing consistency becomes increasingly challenging. The thickness of individual functional regions, interfacial adhesion, spatial positioning, and component distribution can all introduce additional sources of variability. In particular, the interfacial definition and relative layer thicknesses of Janus structures, interlayer diffusion and bonding stability in multilayer systems, as well as rheological behavior, printing parameters, and structural fidelity during 3D printing, all require systematic batch-to-batch validation [38,133,134]. Therefore, translational studies of antibacterial hydrogels should not be limited to demonstrating whether a laboratory formulation can simply be “scaled up.” Instead, development should follow a Quality by Design (QbD) framework, in which polymer molecular weight, degree of substitution, raw-material purity, and characteristics of functional components are considered potential critical material attributes (CMAs), while mixing, crosslinking, homogenization, printing, and curing conditions are defined as critical process parameters (CPPs). On this basis, critical quality attributes (CQAs) directly associated with clinical performance should be established, including compositional uniformity, gelation time, degree of crosslinking, mechanical properties, swelling and degradation behavior, drug loading, release kinetics, antibacterial activity, and spatial structural consistency.

In addition, sterilization and shelf-life stability are critical issues that should be considered early in the product development of antibacterial hydrogels intended for clinical translation. Owing to their high water content and crosslinked polymer networks, hydrogels are particularly sensitive to thermal, radiation-based, and chemical sterilization conditions. Sterilization should therefore not be regarded merely as an independent post-fabrication step, but rather as a critical processing operation that may alter the final properties of the product [135,136]. For example, high-temperature steam sterilization may promote hydrolysis, dehydration, or network rearrangement of certain polymer chains. Ionizing radiation, such as γ-irradiation and electron-beam irradiation, can generate free radicals and, depending on polymer composition, irradiation dose, and hydration state, induce either chain scission or additional crosslinking. Although ethylene oxide (EtO) is suitable for some heat-sensitive materials, it can react with functional groups such as amino, carboxyl, thiol, and hydroxyl groups, while residual EtO and its by-products also require careful control [135,136,137,138]. These molecular- and network-level alterations may subsequently change crosslinking density, pore architecture, and hydrophilicity, thereby affecting hydrogel swelling capacity, mechanical strength, degradation rate, and drug-release kinetics. In stimuli-responsive or sensing antibacterial hydrogels, sterilization may additionally alter temperature-, pH-, light-, electrical-, or strain-responsive behavior, as well as signal stability [136,137,138,139]. Therefore, retention of an intact macroscopic appearance after sterilization cannot by itself be taken as evidence that the therapeutic functions of the hydrogel remain unchanged. This issue is particularly important for hydrogels incorporating bioactive components such as proteins, growth factors, enzymes, extracellular vesicles/exosomes, or platelet-rich plasma (PRP), because these components are generally more susceptible than the polymeric matrix to heat, irradiation, chemical reactions, storage conditions, and repeated freeze–thaw cycles. Such stresses may lead to protein denaturation, loss of biological activity, vesicle aggregation or membrane disruption, and consequent functional deterioration [138,140,141]. Accordingly, for complex bioactive hydrogels that cannot tolerate terminal sterilization, the suitability of terminal sterilization should be carefully weighed against aseptic-processing strategies based on sterile raw materials, aseptic formulation, and aseptic filling [135].

## 5. Conclusions

Antibacterial hydrogels are evolving from materials primarily designed for pathogen elimination toward adaptive therapeutic platforms that coordinate infection control, microenvironment regulation, and tissue regeneration. Owing to their highly hydrated three-dimensional networks, tunable crosslinking structures, and strong capacity for incorporating functional components, antibacterial hydrogels can provide localized coverage at infected sites, retain active agents, and enable sustained release while simultaneously supporting antibacterial activity, mechanical adaptation, and tissue repair. Their design has gradually evolved from the simple loading of individual antibacterial agents toward the coordinated regulation of antibacterial mechanisms, network architecture, pathological microenvironments, and tissue-regenerative processes. Cationic polymers, metal ions, N-halamines, nanozymes, and photothermal materials can exert antibacterial effects through membrane disruption, metabolic interference, oxidative damage, and thermal injury. The integration of multiple mechanisms can enhance the eradication of drug-resistant bacteria and biofilms. However, excessive metal-ion release, ROS accumulation, and local overheating may also damage healthy tissues. Therefore, material evaluation should not rely solely on short-term bactericidal efficiency but should also comprehensively consider effective dosage, duration of action, biocompatibility, and in vivo repair outcomes. With respect to structural construction, natural polymer scaffolds, hierarchical pore architectures, multiple crosslinking, and dynamic networks can improve the mechanical stability, mass-transfer efficiency, self-healing capability, and wet adhesion of hydrogels. Stimuli-responsive strategies, three-dimensional printing, and Janus and core–shell architectures further enable stage-specific release of active components and spatial compartmentalization of functions. Nevertheless, in vitro response profiles do not necessarily translate into precise regulation in vivo, because complex body fluids, proteins, ions, and mechanical motion may alter material release, adhesion, and sensing behavior. Existing sequential therapeutic and theranostic systems also predominantly exhibit stage-dependent functional preferences or quasi-closed-loop operation following a “detection–manual assessment–external intervention” process, rather than genuinely autonomous therapeutic feedback.

Future research should establish standardized antibacterial evaluation frameworks, strengthen the time-resolved characterization of component release and migration, and provide more rigorous validation of the underlying mechanisms of action. The long-term biosafety of metal ions, nanozymes, photothermal agents, and conductive fillers should also be systematically assessed. Future antibacterial hydrogel design should therefore move beyond the simple accumulation of functions toward quantitative coordination among material structure, pathological signals, release behavior, and therapeutic outcomes. Equally important, translational development should incorporate clinically relevant polymicrobial and chronic-wound models, systematic long-term biosafety assessment, batch-to-batch manufacturing control, sterilization compatibility, and functional shelf-life validation. Standardized quality attributes and clinically meaningful endpoints will be essential for determining whether the performance observed in laboratory studies can be reproduced across manufacturing batches and translated into consistent therapeutic benefit in patients. Ultimately, the next generation of antibacterial hydrogels should not be defined merely by the number of integrated functions, but by their ability to deliver reproducible, stage-appropriate, safe, and clinically validated treatment across the continuum from infection control to tissue regeneration.

## Figures and Tables

**Table 1:** Comparison of the construction mechanisms and performance of antibacterial hydrogels with multiple crosslinking.

Hydrogel System	Network Construction Mechanism	Antibacterial Mechanism	Representative Performance	Reference
PVA–PHMG–ADSP	Freeze–thaw physical crosslinking and multiple hydrogen bonds	Cationic guanidinium groups of PHMG disrupt bacterial membranes	Fracture stress, 390 kPa; fracture strain, 336%; compressive stress, 10.26 MPa; conductivity, 1.25 mS·cm^−1^	[84]
PVA/SA/dopamine/borax/TA	Microcrystalline crosslinking, dynamic boronate ester bonds, and hydrogen bonds	TA chelates iron and disrupts cell-wall integrity	Tensile strength, 4.06 MPa; elongation at break, 569.12%	[85]
AHEC/OGG–PDA/Zn^2+^/MWCNTs-SH	C–S covalent bonds, Schiff-base bonds, and metal coordination	Zn^2+^ disrupts bacterial membranes and induces oxidative stress, while MWCNTs-SH provides synergistic antibacterial activity	Covalent–dynamic coordination double network combining structural stability with reconfigurability	[86]
PVA/PA–Fe	Crystalline crosslinking, hydrogen bonds, and Fe^3+^ coordination	Synergistic antibacterial activity of PA and Fe^3+^	Tensile strength, 1.22 MPa; toughness, 1.82 MJ·m^−3^; strength retention after 36 h of water immersion, 67.96%	[87]
Q-TA/NiAm@SI-CNC	Covalent–coordination double network and sacrificial bonds	Quaternary ammonium groups disrupt bacterial membranes, while TA/Al^3+^ provides synergistic antibacterial activity	Improved dispersibility, stability, and toughness	[88]
3-FPBA/tobramycin/TA	Dynamic imine bonds, boronate ester bonds, and N–B coordination	Temperature-responsive tobramycin release	Gel-to-sol transition at approximately 40 °C; accelerated drug release at 45 °C	[89]

**Table 2:** Comparative antibacterial performance, biocompatibility, and key trade-offs of representative antibacterial hydrogel systems.

Hydrogel System	Antibacterial Performance	Biocompatibility	Limitations	Reference
CS/TA hydrogel	~94% inhibition against *S. aureus* and >80% against *E. coli* at the highest TA content	Hemolysis of CS/TA-4 exceeded the 5% safety threshold	Increasing TA content enhances antibacterial activity but may reduce hemocompatibility	[53]
AMCB-FTB smart hydrogel	High antibacterial efficacy reported through multimodal synergistic therapy	Later-stage ROS scavenging and O_2_ generation are designed to reduce oxidative stress	Complex multicomponent design; catalytic efficacy depends on wound pH and local chemical conditions	[62]
PEDOT@PBT-PSBMA/SF hydrogel	99.02% against *E. coli*; 99.14% against *S. aureus*; 97.70% in vivo against MRSA	Wound temperature maintained at ~45 °C to limit thermal injury	Requires NIR irradiation; treatment outcome depends on irradiation parameters	[70]
GOx-modified Exos-loaded hydrogel	>90% bactericidal efficiency against both strains	Promoted angiogenesis, collagen deposition, and M2 macrophage polarization	GOx-generated H_2_O_2_ is beneficial for antibacterial activity but excessive ROS generation may damage host tissue	[94]
NiCo_2_O_4_/ZnO bilayer Janus hydrogel	>95% inhibition against both strains	NiCo_2_O_4_-mediated ROS scavenging reduces oxidative stress	metal-ion dose and long-term inorganic-material biosafety require further assessment	[102]
ROS-responsive PRP-loaded double-network hydrogel	>99% inhibition against both strains	Reduced IL-1β, increased IL-10, CD31, and α-SMA expression	Multicomponent formulation and stage-dependent release increase design complexity	[107]

Note: Antibacterial efficiencies are values reported in the original studies and should not be interpreted as directly equivalent across systems because experimental conditions, including bacterial strains, inoculum concentrations, exposure times, antibacterial assays, and external-stimulation parameters, differed among studies. Biocompatibility and safety information is summarized according to the assays reported in the original studies. “Not specified” indicates that quantitative data were not provided in the present review and should not be interpreted as evidence of poor biocompatibility.

## Data Availability

No new data were created or analyzed in this study. Data sharing is not applicable to this article.
